# Factors associated with nurses’ attitudes for providing oral care in geriatric care facilities: a cross-sectional study

**DOI:** 10.1186/s12903-023-03517-7

**Published:** 2023-10-26

**Authors:** Mengxia Chen, Yanqiu Weng, Jingwen Zhang, Liyan Gu, Wenyao Chen, Mengting Qiao, Mengdi Wang, Xiaorong Huang, Lan Chen, Lingjuan Zhang

**Affiliations:** 1 Education and Scientific Research Department of Clinical Nursing, The First Affiliated Hospital of Naval Medical University, Shanghai, China; 2 Shanghai Quality Control Center of Geriatric Care, Shanghai, China; 3 Department of Neurology, No. 905 Hospital of PLA Navy, Shanghai, China; 4 Department of Nursing, Huzhou University, Huzhou, Zhejiang China; 5 Department of Nursing, Shanghai General Hospital, Shanghai Jiao Tong University School of Medicine, Shanghai, China

**Keywords:** Nurse, Attitude, Oral hygiene, Geriatric care facilities

## Abstract

**Background:**

The world’s population is getting older. This issue is accompanied by a rise in the number of older people suffering from dementia and disability, for whom oral hygiene care is challenging. Nurses’ attitudes toward providing oral care (POC) are critical for the elderly, while few studies have investigated the determinant factors of nurses’ attitudes by identifying the current work pressure, resilience and self-efficacy in geriatric care facilities (GCFs). It is of great significance to explore the nurses’ attitudes toward POC and associated influencing factors related to psychological aspects including resilience, self-efficacy, and stress from the workplace.

**Methods:**

Attitudes for Providing Mouth Care (A-PMC) in Chinese version were used in this cross-sectional study with 160 nurses in 2 GCFs. Data were collected using online questionnaires and analyzed by multiple linear regression analysis. Statistically significant values were considered at p < 0.05.

**Results:**

A total of 160 nurses participated in this study, with an average age of 32.86 ± 7.43. The mean score for the A-PMC was 2.81 ± 0.47. The score of A-PMC was negatively correlated with work pressure (r=-0.332, p < 0.01), and positively correlated with resilience (r = 0.735, p < 0.01) and self-efficacy (r = 0.425, p < 0.01) respectively. Multiple linear regression analyses identified that the potential influencing factors of A-PMC were education background, work hours every shift, self-efficacy, work pressure and resilience.

**Conclusions:**

The study results indicate nurses’ attitudes regarding PMC were at a low level, which is influenced by many factors. To improve nurses’ attitudes toward PMC and the oral hygiene (OH) of the elderly in GCFs, it is necessary to increase nurses’ education and training, establish a reasonable and effective incentive mechanism to improve nurses’ work motivation and other intervention measures to reduce work pressure.

**Supplementary Information:**

The online version contains supplementary material available at 10.1186/s12903-023-03517-7.

## Background

Population ageing is one of the major social transformations of the twenty-first century, the number of elderly people continues to grow worldwide. As of 2022, the global population aged 65 and above has reached 771 million [1], China’s population aged 60 and above has reached 280 million, and the population aged 65 and above has reached 210 million [2]. Ongoing demographic changes will lead to a surge in demand for care among the elderly. Due to resource constraints and the impact of the empty-nested phenomenon, many frail or functionally dependent older people (DOPs) are housed in GCFs [3].

Poor oral health is a major public health problem [4]. Elderly people living in GCFs for long periods are often accompanied by varying degrees of physical and cognitive impairment, and their oral health problems are becoming increasingly prominent due to aging oral function and the coexistence of various chronic diseases, which will further lead to a reduction in eating and affect quality of life.

Daily oral care (OC) is the most important factor contributing to good OH. Nurses with a positive attitude toward POC play a crucial role in improving older people’s oral health and well-being [5]. The actual situation is that nursing staff tend to pay more attention to the basic diseases of the elderly, and the oral problems of the elderly do not receive timely and effective intervention [6, 7] Hence, Changing nurses’ attitudes for the benefit of residents’ oral health and OH has been the primary goal of numerous research [8, 9]. Our previous research also took the initiative to analyze nurses’ oral care attitudes and self-efficacy in GCFs in China and to analyze differences across facilities, and nations [10]. Other research has focused on older people’s existing dental diseases and barriers and facilitators perceived by various healthcare professionals [11–15], with little study of nurses’ attitudes from their perspective. So the primary psychological and character-impacting variables are yet unclear.

Nurses working in GCFs are under increasing pressure as the population ages. A previous study has found that nursing tasks are numerous and strong role pressure could decrease nurses’ job expectancies, influencing their work attitudes and efficiency [7]. Furthermore, Psychological resilience is defined as a positive response to adversity (including work pressure), and several studies have been conducted to investigate the association between work pressure and psychological resilience in nurses [16]. Nurses with strong self-efficacy have greater levels of engagement at work and believe they can complete their jobs and deal with potential challenges sufficiently and effectively [17].

From the perspective of nurses’ psychology and work-related stress, this study aims to investigate whether these factors influence nurses’ attitudes about POC to the old, to understand their beliefs and attitudes, work pressure, and resilience, which are especially significant since they relate to care provision and, ultimately, patients’ OH. This gap cloud be filled in our study by understanding the factors that influence nurses’ attitudes, which will also assist nursing administrators in providing evidence for care promotion programs and improving the OH of the elderly.

## Method

### Study design and participants

An online cross-sectional study was carried out with the A-PMC from April 01 to April 30, 2023, in Shanghai, China. This study used a convenience sampling method and 160 nurses in 2 GCFs were invited to participate voluntarily in this survey. The inclusion criteria for participants were: (a) Registered Nurses in GCFs; (b) informed consent and voluntary participation in this study. However, nurses who did not work in GCFs during the survey period were excluded (off-site training or sick leave).

### Sample size

We selected 11 possible influencing factors through a literature review. According to the principle that the sample size should be 5 to 10 times the number of independent variables, the estimated minimum sample size was 110; considering a likely attrition rate of 10% and sampling error, the appropriate recommended sample size was at least 122, and we eventually collected data from 160 participants in 2 GCFs.

### Questionnaire design

#### Demographic information:

age, sex, marital status, child-rearing, educational background, professional title, working experience, average working hours per day, and the number of patients in charge every day.

*A-PMC* and *SE-PMC scale*: The A-PMC and SE-PMC scales were developed by Wretman in 2020 [18]. The Chinese Version was translated, culturally adapted and psychometrically tested in our previous study [19]. A-PMC(11 items) has 2 factors: Care of Residents’ teeth (CRT) and “Care of Own teeth(COT). SE-PMC(11 items) has 3 factors: Promoting Oral Hygiene (POH), Providing Mouth Care (PMC) and Obtaining Cooperation (OC). Both SE-PMC and A-PMC scales adopted Likert 4-level scoring method, with scores ranging from “Strongly Disagree”, “Not Quite Agree”, “Agree”, and “Strongly Agree” assigned 1–4 points, with a maximum of 44 points. The higher the scale score, the higher the nurses’ attitude and self-efficacy towards POC. The two scales are self-rating scales with a first-person perspective. In the present study, Cronbach’s alpha coefficient for the A-PMC and SE-PMC were 0.995 and 0.998 respectively.

*Connor-Davidson Resilience Scale(CD-RISC)*: CD-RISC is a self-rating measure that examines resilience to both social and non-social causes of adversity. CD-RISC includes 5 factors: personal competence, tolerance of negative emotions, acceptance of change, sense of control, and spiritual belief. CD-RISC consists of 25 items that may be rated on a five-point scale (0 = not true at all, 1 = seldom true, 2 = sometimes true, 3 = often true, 4 = true nearly all the time); a high score indicates stronger resilience [20]. The Chinese version of CD-RISC has high validity and reliability among Chinese people [19, 21] and Cronbach’s alpha of CD-RISC in this study is 0.978.

*China Nurses’ Work Stress Scale(CNSS)*: Li [22] developed the CNSS based on the Nursing Work Stress Scale [23], a Chinese scale intended specifically for nurses to analyze their stress status. CNSS is comprised of 35 items and 5 dimensions: (1)nursing professional and work problems; (2) stress in time allocation and workload;(3) stress in working environment and equipment; (4) stress in patient care; and (5) stress in management and interpersonal relationship. CNSS is rated on a four-point scale (1 = no stress, 2 = mildly stressful, 3 = moderately stressful, 4 = more stressful), with higher scores indicating more stress. In this study, Cronbach’s alpha for CNSS is 0.969.

### Data collection

The questionnaire was created using “Wenjuanxing” (https://www.wjx.cn/), an electronic questionnaire platform. WeChat (a social media app) was used to send a web page of the questionnaire to participants’ mobile phones. To avoid missing things, all questions were made necessary. Before distributing the questionnaire, we contacted the nurse directors of 2 GCFs and explained the purpose of this study to obtain their permission. The inquiry was kept private and anonymous. Respondents could amend their answers by clicking the “Back” button at the bottom of each page, a feature offered by the Wenjuanxing platform. Participants might exit the survey at any moment by closing the link or not submitting the poll, and their data would not be saved. The questionnaire could not be withdrawn after it was submitted and each WeChat account can only fill in the questionnaire once to avoid duplicate submissions. There were no missing items among the 160 questionnaires submitted, although one was invalid (option selection all “1”). As a result, 159 surveys were valid, with an effective response rate of 99.4%.

### Data analysis

SPSS version 26.0 software was used for statistical analysis. Continuous variables were expressed in mean (M) ± standard deviation (SD), whereas categorical variables were described in number and frequency. To determine the proper statistical test, distributions were examined for normality. The independent sample t-tests or analysis of variance were used to compare means based on the results. Statistical significance was assessed using ANOVA with LSD post hoc test. Then the variables with *p* < 0.05 in the univariate analysis are included in the multiple linear regressions. Pearson’s correlation test was used to examine the relationship between variables. The data distribution meets the standard assumptions underlying regression models. So influencing factors of A-PMC were determined by multiple linear regressions. The thesis brings the categorical variable (education level and number of patients in charge per shift) as dummy variables into the regression analysis model. No missing data were found during the analysis due to the prior setting of the online survey design. The statistical significance level was set at < 0.05.

To determine the factors independently related to A-PMC, multiple regression analyses were conducted. The mean score of A-PMC was considered as the dependent variable, while the education background, whether had children or not, the number of patients in charge, average working hours per day, and the scores of SE-PMC, CD-RISC, and CNSS were considered as predictor variables. To ensure collinearity was not an issue, variance inflation factors (VIF) and tolerances were used to check all models. The VIF values were less than 5 and the tolerance values were greater than 0.1, indicating no presence of multicollinearity.

### Ethical considerations

On the first page of the survey, accessed by scanning the QR code, we offered an introduction to the survey as well as informed permission. If participants were interested in looking through the survey questionnaire, they would click the box on the first page that said “I agree to participate in this research of my own volition”. The Wenjuanxing platform automatically logged their informed consent, indicating that the participants had provided informed consent. They would then participate in the official survey. According to the Declaration of Helsinki, the poll guaranteed autonomy, confidentiality, and no damage. Ethical approval was obtained from the Shanghai Changhai Hospital Ethics Committee (CHEC2023-066).

## Results

### Demographic characteristics of participants

All nurses were female (n = 159) with an average age was (32.86 ± 7.43). The proportion of nurses with bachelor’s degrees was 75.47% (n = 120). More than half have less than 10 years length of employment in GCFs. 57.86% (n = 92) nurses work 6 ~ 8 h per shift and 46.54% (n = 74) nurses take charge of 6 ~ 10 patients every day. Table 1 displays the demographic profiles of nurses in GCFs.

Table 1 shows the difference in A-PMC scores based on their demographic characteristics. The study found that nurses in GCFs with higher education, Shorter work hours every day and fewer patients in their charge per day had higher scores on the A-PMC assessment.

**Table 1:** Social demographic data of the sample (n = 159)

Variables		Frequency(%)	A-PMC	*F/t*	*P*	*LSD*
Age (years)				1.807	0.168	
	20–30	69(43.49)	2.73 ± 0.46			
	31–40	69(43.39)	2.88 ± 0.50			
	41–60	21(13.21)	2.86 ± 0.40			
Marital status				1.091	0.338	
	Married	118(74.21)	2.85 ± 0.49			
	Single	34(21.38)	2.72 ± 0.42			
	Divorced	7(4.40)	2.73 ± 0.39			
Had one or more children				0.027	0.869	
	Yes	99(62.26)	2.81 ± 0.46			
	No	60(37.74)	2.82 ± 0.53			
Education				7.855	**0.001**	
	1.secondary vocational education	4(2.52)	2.59 ± 0.38			
	2.higher vocational education	35(22.01)	2.56 ± 0.42			2<3^*^
	3.university education	120(75.47)	2.90 ± 0.47			
Length of work in GCF(years)				1.554	0.203	
	0–10	89(55.97)	2.76 ± 0.48			
	11–20	60(37.74)	2.87 ± 0.44			
	21–30	8(5.03)	3.01 ± 0.44			
	>31	2(1.26)	2.45 ± 0.51			
Professional title				0.259	0.772	
	Junior	89(55.97)	2.84 ± 0.50			
	Intermediate	68(42.77)	2.78 ± 0.45			
	Senior	2(1.26)	2.82 ± 0.00			
Average working hours per day				3.543	**0.031**	
	1. 6 ~ 8 h	92(57.86)	2.90 ± 0.48			1>2^*^
	2. 8 ~ 10 h	62(38.99)	2.71 ± 0.45			
	3.>10 h	5(3.14)	2.64 ± 0.29			
Number of patients in charge per day				4.572	**0.004**	
	1. ≤6	23(14.47)	3.07 ± 0.55			1>2^*^, 1>3^*^, 1>4^*^ 2>4^*^
	2. 6 ~ 10	74(46.54)	2.85 ± 0.51			
	3. 10 ~ 15	22(13.83)	2.75 ± 0.40			
	4.>15	40(25.16)	2.64 ± 0.29			

*means p<0.05

### A-PMC and other variables in this study

The study found that the mean score of A-PMC was (2.81 ± 0.47). Furthermore, the mean score for the attitude towards taking care of their own teeth was (3.46 ± 0.51), while the mean score for the attitude towards taking care of the teeth of elderly people in GCFs was (2.28 ± 0.77).In addition, the mean scores for CD-RISC and CNSS are 64.48 (SD = 16.85) and 65.52(SD = 14.06), respectively. All are shown in Table 2.

**Table 2:** Descriptive statistics for all scales in this survey(n = 159)

Scale	Theoretical range	Mean	SD
A-PMC	1–4	2.81	0.48
SE-PMC	1–4	3.03	0.42
CD-RISC	0-100	64.48	16.85
CNSS	35–140	65.52	14.06

### Relationship between A-PMC, SE-PMC, CD-RISC and CNSS

The results of the Pearson correlation analysis demonstrate a positive relationship between A-PMC, SE-PMC, and CD-RISC while showing a negative relationship with CNSS. In addition, there is a significant positive correlation between SE-PMC and CD-RISC. As illustrated in Table 3.

**Table 3:** Correlation between A-PMC and SE-PMC, CD-RISC and CNSS among nurses in GCFs

Variables	A-PMC	SE-PMC	CD-RISC	CNSS
A-PMC	1			
SE-PMC	0.425**	1		
CD-RISC	0.735**	0.368**	1	
CNSS	-0.332**	0.037	0.010	1

**p <0.01

### Multiple linear regression of factors affecting A-PMC of nurses in GCFs

The A-PMC regression model yielded significant results (F = 109.817, p < 0.001), with an adjusted coefficient of determination (Adj R^2^) of 0.828. Notably, education background (β = 0.256, p < 0.001), the number of patients in charge (β = -0.265, p < 0.001), SE-PMC (β = 0.154, p < 0.001), CD-RISC (β = 0.468, p < 0.001), and CNSS (β = -0.280, p < 0.001) were found to be significant factors associated with A-PMC. As is shown in Table 4.

**Table 4:** Multiple linear regressions of independent variables on A-PMC

Variables	B	SE	β	*t*	*p*
Constant	0.891	0.191		4.674	<0.001
SE-PMC	0.172	0.040	0.154	4.264	<0.001
CD-RISC	0.329	0.034	0.468	9.700	<0.001
CNSS	− 0.207	0.025	− 0.280	-8.122	<0.001
Education level Reference: secondary vocational education					
higher vocational education	0.329	0.120	0.289	2.740	0.007
university education or above	0.528	0.115	0.481	4.585	<0.001
Number of patients in charge per shift	− 0.123	0.016	− 0.265	-7.865	<0.001
Reference: ≤6 patients					
6 ~ 10 patients	− 0.088	0.054	− 0.093	-1.642	0.003
10 ~ 15 patients	− 0.146	0.067	− 0.106	-2.167	0.032
>15 patients	− 0.338	0.059	− 0.310	-5.766	<0.001

## Discussion

The oral health of elders has received more attention in recent years [24]. In this study, the mean score of A-PMC in GCFs was 2.81 ± 0.47, which means nurses had an unfavourable attitude towards POC in their nursing care plan. The possible explanation may be that the burden increases as the number of older individuals in GCFs increases. As a result, nurses may feel severe stress, which may impair their degree of professional engagement, as well as the quality of patient treatment [25], and may eventually affect POC attitudes. The result of this study was lower than the results of the Shanghai Quality Control Center of Geriatric Care research in 2021(2.98 ± 0.35) [17]. This might be because participants were recruited from two GCFs in Shanghai, resulting in a small sample size; additionally, due to a shortage of nurses, combined with the short pre-job education and training time, this may resulted in low attitudes to POC. Besides, 2 GCFs participating in this study have been in operation for more than 10 years, implying that the infrastructure, staffing, and standardized procedures are poorer than in newly founded GCFs, which may result in a lower score.

The examination of discrepancies in A-PMC among different demographic characteristics has proved that nurses with high education have high A-PMC (*p* < 0.05). The study by Lina [26] noted that the most common barriers to the POC included a lack of education and training in oral health, and caregivers expressed a desire to increase their knowledge of oral care and to update their knowledge regularly to improve POC. This might be due to knowledge differences among nurses: the higher the educational background, the better knowledge, and the more positive attitude toward the disease.

Besides, some guidelines also suggest that guidance for oral health education for nurses and caregivers should be provided at the institutional level, improvements in knowledge might be obtained by just performing education, which is consistent with our findings [12, 27]

The influence of the length of work experience on nurses’ attitudes regarding POC in this study was consistent with Chen [28] and Wretman [18], with no significant changes. Interestingly, Chen’s study found that nurses over the age of 34 had higher attitude scores than younger nurses [28], whereas this study found that nurses in the 31–40 age group had higher attitudes toward POC than the other groups, which may be due to the small population of nurses over the age of 41 in this study.

Multiple regression analysis further revealed a positive association between education background and A-PMC, demonstrating that as nurses’ education improves, so do their attitudes, and the result is consistent with a previous study which found that educational level was significantly associated with the level of knowledge about dental infection [29], and knowledge would help to understand the importance of oral health care [30]. Possible explanations for this are that nurses with higher education have received more knowledge about oral care and understand the importance of oral care as well as the potentially serious consequences of poor OH; additionally, nurses with a bachelor’s degree or higher have a significant advantage in critical thinking when compared to nurses with lower education. Therefore, it is recommended that nurses should attend educational sessions in the form of seminars, and workshops to improve their attitude and practice. This will increase understanding and develop a favourable attitude toward dental health care practice.

The number of patients charged by nurses and their attitude toward POC were shown to have a negative association in this study. The number of patients in charge means the consequent stress and huge workload and may directly affect their work engagement [31], which is significant for patient outcomes [32]. An increase in daily workload requires performing more nursing tasks, which finally leads to poor motivation for nurses to offer dental care. Hence, GCFs should optimize shift scheduling and flexible work arrangements, hire more nurses if necessary, and lower nurses’ workloads. As the literature mentioned, nurses may have higher self-efficacy and a more positive attitude in a pleasant and relaxing working environment [33, 34].

Nurses’ self-efficacy in POC is positively connected to their attitude. One possible reason is that self-efficacy influences nurses’ work initiative and problem-solving abilities [35]. Nurses with a high sense of self-efficacy are more likely to have favourable attitudes regarding POC because they pay greater attention to the quality of oral care and monitor the results of oral interventions. Besides, self-efficacy was positively correlated with psychological resilience in this study, as confirmed in previous studies [36, 37].

This study discovered a negative relationship between stress and A-PMC. This might be because nurses working in GCFs experience more pressure from families, patients, and society, resulting in a lack of professional identity and negative attitudes. According to previous studies, poor work environments make it difficult for nurses to perform their professional roles [38].In addition, the greater her psychological resilience, the higher the total score of A-PMC. A good psychological level will also help nurses understand problems correctly, solve problems more actively, and thus work more actively and effectively.

This study’s sample was restricted to two GCFs in Shanghai, which introduces some bias. In addition, self-reported data were collected which possibly led to measurement subjectivity because of recall bias. Future research on nurses’ attitudes toward delivering dental care can widen the sample collection’s purview for validation and further investigate the association between the contributing elements.

## Conclusion

The role of nurses in maintaining OH and well-being is undeniable. We concluded that nurses’ attitudes toward POC were relatively less positive. This finding may be related to psychological aspects including low resilience, low self-efficacy, and heavy stress from the workplace. We believe that our findings will help nurse managers better understand the factors affecting nurses’ attitudes toward POC and allow them to determine the primary strategies to address these factors, thereby enhancing OH in GCFs.To change the attitude of the nurses, it is advised that GCFS can enhance their practice by providing training and equipping the health care setup (like integrated dental providers). Besides, setting up a fair and efficient reward system to boost nurse motivation and increase humanistic care to improve the OC.

### Electronic supplementary material

Below is the link to the electronic supplementary material.

Supplementary Material 1

## Data Availability

Data are available upon reasonable request. Data presented in this study are available on request from the corresponding author.

## List of abbreviations

POC: Providing Oral Care
GCFs: Geriatric Care Facilities
A-PMC: Attitudes for Providing Mouth Care
OH: Oral Hygiene
DOP: Dependent Older People
WHO: World Health Organization
OC: Oral Care
SE-PMC: Self-Efficacy for Providing Mouth Care
CD-RISC: Connor-Davidson Resilience Scale
CNSS: China Nurses’ Work Stress Scale
